# Comparative Saturation Binding Analysis of ^64^Cu-Labeled Somatostatin Analogues Using Cell Homogenates and Intact Cells

**DOI:** 10.1021/acsomega.3c02755

**Published:** 2023-06-22

**Authors:** Martin Ullrich, Florian Brandt, Reik Löser, Jens Pietzsch, Robert Wodtke

**Affiliations:** † Helmholtz-Zentrum Dresden-Rossendorf, Institute of Radiopharmaceutical Cancer Research, Bautzner Landstraße 400, Dresden 01328, Germany; ‡ School of Science, Faculty of Chemistry and Food Chemistry, Technische Universität Dresden, Mommsenstraße 4, Dresden 01069, Germany

## Abstract

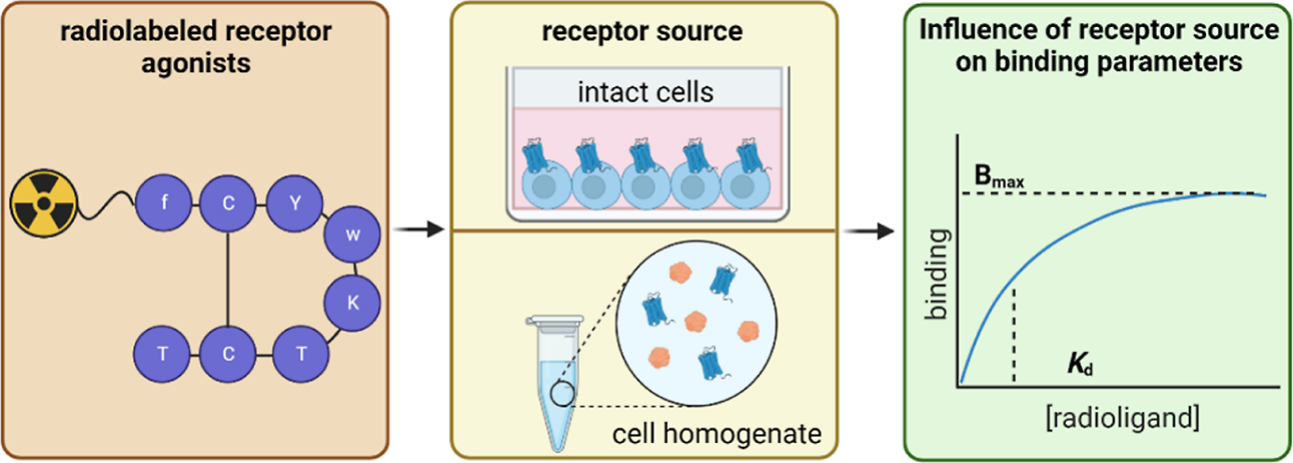

The development of novel ligands for G-protein-coupled receptors (GPCRs) typically entails the characterization of their binding affinity, which is often performed with radioligands in a competition or saturation binding assay format. Since GPCRs are transmembrane proteins, receptor samples for binding assays are prepared from tissue sections, cell membranes, cell homogenates, or intact cells. As part of our investigations on modulating the pharmacokinetics of radiolabeled peptides for improved theranostic targeting of neuroendocrine tumors with a high abundance of the somatostatin receptor sub-type 2 (SST_2_), we characterized a series of ^64^Cu-labeled [Tyr^3^]octreotate (TATE) derivatives in vitro in saturation binding assays. Herein, we report on the SST_2_ binding parameters measured toward intact mouse pheochromocytoma cells and corresponding cell homogenates and discuss the observed differences taking the physiology of SST_2_ and GPCRs in general into account. Furthermore, we point out method-specific advantages and limitations.

## Introduction

G-protein coupled receptors (GPCRs) are one of the most important pharmacological targets not only due to their involvement in a plethora of physiological processes but also due to an often well-defined and cell surface-exposed ligand binding site that enables the design of target molecules.^1,2^ Moreover, GPCRs are attractive for the development of targeted radiopharmaceuticals for the diagnosis and therapy of tumors with radiolabeled agonists of the somatostatin receptor sub-type 2 (SST_2_) as probably the most prominent examples.^3^ In this context, **[**^**177**^**Lu]Lu-DOTA-TATE** represents the first radiopharmaceutical for peptide receptor radionuclide therapy of neuroendocrine tumors, which has been approved by the EMA and FDA.^4^ For the development of novel ligands targeting GPCRs including the elucidation of their pharmacological effects, initial characterization via binding and functional assays is necessary.^5^ While the assessment of functional effects on cells upon ligand binding implies the use of intact cells (whole-cell assay format), binding studies, typically with the use of radioligands or fluorescent probes, are performed with different sources of the receptor, including tissue sections, membrane preparations, cell homogenates, and also intact cells.^6−12^ In this context, there is a controversy about which of the receptor sources should be preferred with regard to the significance of the determined binding parameters, in particular when comparing cell homogenates and intact cells.^13−15^

Based on the vector molecule [Tyr^3^]octreotate (TATE or TOCA) for SST_2_ targeting, we recently reported on novel TATE derivatives modified with albumin binders (**[**^**64**^**Cu]Cu-NODAGA-cLAB-TATEs**^16^), cleavage sequences for neprilysin, or both (**[**^**64**^**Cu]Cu-NODAGA-NES-TATEs**^17^) to systematically explore the suitability of these structural modifications for modulating the pharmacokinetic properties of peptidic radioligands. As part of the in vitro radiopharmacological characterization, the SST_2_ affinity of the **[**^**64**^**Cu]Cu-NODAGA-cLAB-TATEs** was characterized in saturation binding analyses using cell homogenates of mouse pheochromocytoma cells (MPC) exhibiting high levels of SST_2_.^18^ For investigating the **[**^**64**^**Cu]Cu-NODAGA-NES-TATEs**, we switched to intact cells instead of cell homogenates, primarily due to an occasionally high nonspecific binding when using cell homogenates. Having both methods established, we subsequently characterized a set of ten ^64^Cu-labeled TATE derivatives comparing both MPC cell homogenates and intact cells. Herein, we report on the binding parameters obtained with the two different saturation binding assays and discuss the results considering the different assay formats when using cell homogenates and intact cells, the physiology of GPCRs, as well as the inherent limitations for determining the binding affinity of GPCR ligands.

## Results and Discussion

**Figure 1:**
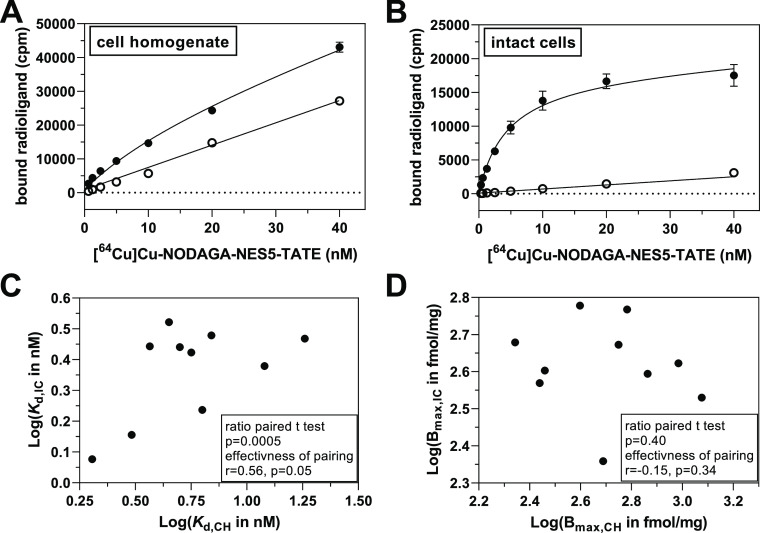
Comparison of saturation binding data using cell homogenates (CH) and intact cells (IC) (A/B) saturation binding of **[**^**64**^**Cu]Cu-NODAGA-NES5-TATE** toward MPC cell homogenates (A) and intact cells (B) with data for total and nonspecific binding (in the presence of 1 μM DOTA-TATE) shown as filled and open circles, respectively. Plot in B was published recently by us (Adapted with permission from Brandt et al.^17^ Copyright 2023, American Chemical Society). Regression analysis was performed by using the model of “one site—total and nonspecific binding” as implemented in GraphPad Prism (Version 9.4.1). (C,D) Correlation plots for the ratio paired t tests of *K*_d_ (C) and *B*_max_ (D) data with results of the tests given in the boxes.

For a series of ten previously described ^64^Cu-labeled TATE derivatives (Table 1), total and nonspecific binding to intact cells and cell homogenates were assessed over a range of radioligand concentrations. Exemplary saturation binding curves for one radioligand, **[**^**64**^**Cu]Cu-NODAGA-NES5-TATE**, are shown in Figure 1A,B (for other radioligands, see Figure S1 in the Supporting Information). The calculated binding parameters *K*_d_ and *B*_max_ are summarized in Table 2. For the two assay formats employing cell homogenates and intact cells, radioligand binding was performed for 1 h at 37 °C. Generally, the *K*_d_ values toward both SST_2_ sources were in a similar range but appeared to be systematically shifted to lower values when intact cells were used compared to cell homogenates. In contrast, the *B*_max_ values did not reveal such a trend. This is further exemplified by analyzing the *K*_d_ and *B*_max_ values with a ratio paired *t*-test (Figure 1C/D), which revealed that the logarithm of the *K*_d_ ratios is significantly different from 0 (i.e., the *K*_d_ ratios are different from 1, *p* = 0.0005) but not the *B*_max_ values (*p* = 0.40). The respective *K*_d_ ratios are listed in Table 2. The geometric mean of the *K*_d_ ratio is 2.41 (95% CI of 1.65–3.52). To rationalize the observed *K*_d_ shift, different aspects need to be considered that are discussed in the following sections.

**Table 1:** Summary of SST_2_ Ligands Discussed Herein

compound	Chelator	N-terminal modification						SST_2_ scaffold^a^
**DOTA-TATE**	DOTA							TATE
**NODAGA-TATE**	NODAGA-							TATE
**NODAGA-Pra-PEG2-TATE**	NODAGA-					Pra-	PEG2-	TATE
**NODAGA-cLAB1-TATE**	NODAGA-					Pra(X)^b^-	PEG2-	TATE
	(X = *N*^α^-5-azidopentanoyl-*N*^ε^-4-(4-iodophenyl)butanoyl-d-lysine)							
**NODAGA-cLAB4-TATE**	NODAGA-					Pra(X)^b^-	PEG2-	TATE
	(X = *N*^α^-4-azidobenzoyl-*N*^ε^-4-(4-methylphenyl)butanoyl-l-lysine amide)							
**NODAGA-NES1-TATE**	NODAGA-			Nle-	Val-	Lys(N3)-	PEG2-	TATE
**NODAGA-NES2-TATE**	NODAGA-	PAMBA-		Nle-	Val-	Lys(N3)-	PEG2-	TATE
**NODAGA-NES3-TATE**	NODAGA-	PAMBA-		Gly-	Phe-	Lys(N3)-	PEG2	TATE
**NODAGA-NES4-TATE**	NODAGA-	PAMBA-	d-Arg-	Gly-	Phe-	Lys(N3)-	PEG2	TATE
**NODAGA-NES5-TATE**	NODAGA-	PAMBA-	d-Arg-	Gly-	Phe-	Lys(X)^b^-	PEG2	TATE
	(X = *N*^α^-4-pentynoyl-*N*^ε^-4-(4-iodophenyl)butanoyl-l-lysine)							
**NODAGA-JR11**	NODAGA-							JR11

a TATE represents d-Phe-c(Cys-Tyr-d-Trp-Lys-Thr-Cys)-Thr-OH and JR11 represents Cpa-c(d-Cys-Aph(Hor)-d-Aph(Cbm)-Lys-Thr-Cys)-d-Tyr-NH_2._

b The azide and alkyne functionalized albumin binders were coupled via on-resin CuAAC to the fully protected peptides bearing Pra and Lys(N_3_) residues leading to a 1,4-disubstituted 1*H*-1,2,3-triazole linkage. For the synthesis and analytical data to NODAGA-JR11 see the Supporting Information. Aph, 4-aminophenylalanine; Cbm, carbamoyl; Cpa, 4-chlorophenylalanine; Hor, l-dihydroorotic acid; PAMBA, *p*-aminomethyl benzoic acid; Pra, l-propargylglycine.

**Table 2:** Summary of SST_2_ Binding Data for a Series of ^64^Cu-Labeled TATE Derivatives Using MPC Cell Homogenates and Intact MPC Cells

	MPC cell homogenate		intact MPC cells			
^64^Cu-labeled compound^a^	*K*_d_ (nM)^b^	*B*_max_ (fmol/mg of protein)^b^	*K*_d_ (nM)^b^	*B*_max_ (fmol/mg of protein)^b^	uptake fraction (%)^c^	*K*_d_ ratio^e^
**DOTA-TATE**	2.03 (0.48)	562 (218)	1.19 (0.16)	471 (31)	56	1.70
**TATE**	3.05 (0.16)	965 (582)	1.43 (0.39)	420 (77)	64^d^	2.13
**Pra-PEG2-TATE**	4.48 (0.57)	488 (144)	3.33 (0.83)	229 (89)	48	1.35
**cLAB1-TATE**	6.31 (0.99)	1191 (334)	1.73 (0.35)	339 (73)	66	3.66
**cLAB4-TATE**	3.67 (3.30–4.09)^d^	396 (384–408)^d^	2.77 (0.62)	600 (50)	64	1.32
**NES1-TATE**	6.94 (6.19–7.78)	220 (212–229)	3.01 (0.65)	478 (175)	49^d^	2.31
**NES2-TATE**	18.2 (14.2–23.7)	288 (257–327)	2.94 (0.85)	401 (145)	56^d^	6.19
**NES3-TATE**	5.01 (4.04–6.20)	275 (257–294)	2.76 (2.33–3.25)^d^	371 (354–389)^d^	68^d^	1.82
**NES4-TATE**	5.64 (3.66–8.71)	606 (532–698)	2.65 (1.93–3.64)^d^	586 (532–649)^d^	53^d^	2.13
**NES5-TATE**	12.0 (1.50)	731 (189)	2.40 (1.34)	393 (190)	62^d^	5.01
**JR11**	5.35 (5.06–5.65)	764 (751–778)	8.17 (7.38–9.04)	717 (691–744)	28	0.65

a After ^64^Cu-labeling, the non-labeled ligand was not separated or saturated with ^nat^Cu^2+^.

b Data shown are mean values with calculated confidence interval of 68% of one experiment, which was performed in duplicate or triplicate, or mean values ±SEM of 2–3 or 6 (in case of **[**^**64**^**Cu]Cu-NODAGA-TATE** toward intact MPC cells) experiments, which were also performed in duplicate or triplicate.

c The uptake fraction (“internalized” or not acid releasable radioligand) is expressed as percentage of specific total-bound radioligand (Figure S4 in the Supporting Information).

d Data were previously published by us (adapted with permission from Brandt et al.^16^ Copyright 2022 American Chemical Society and Brandt et al.^17^ Copyright 2023, American Chemical Society).

e Ratio of *K*_d_ (cell homogenate)/*K*_d_ (intact cells). If not otherwise stated, the compounds bear NODAGA as chelator moiety for complexation of copper-64.

### Nonspecific Binding and Stability

As depicted in Figure 1A,B for **[**^**64**^**Cu]Cu-NODAGA-NES5-TATE**, the extent of nonspecific binding to intact cells is reduced compared to the nonspecific binding to cell homogenates. This finding appears consistent among all compounds (Figure S1 in Supporting Information). In this context, it is worth noting that the data for total and nonspecific binding to the receptor sources were corrected for nonspecific binding to the well plates (in case of intact cells) and filter (in case of cell homogenates) as also outlined in the experimental descriptions. However, to validate that the extent of nonspecific binding is not caused by the different assay formats itself (washing in well plates versus filtration), the saturation binding assay was also performed for **[**^**64**^**Cu]Cu-NODAGA-NES5-TATE** with intact cells in suspension under the conditions used for cell homogenates (Figure S2 in Supporting Information). The data indicate that the high nonspecific binding to cell homogenates is indeed caused by the receptor source itself as the nonspecific binding to intact cells under the same technical conditions is significantly lower. We hypothesize that this phenomenon originates from exposing the radioligand to cell components in cell homogenates that the radioligand does not encounter in intact cells due to its limited cell permeability.^14^ This clearly favors the use of intact cells over cell homogenates as receptor source. However, the differences in nonspecific binding may not be responsible for the observed trend in the K_d_ values (e.g., due to potential limitations for data analysis) as radioligands such as **[**^**64**^**Cu]Cu-DOTA-TATE** and **[**^**64**^**Cu]Cu-NODAGA-TATE**, that show a low nonspecific binding for both receptor sources, also exhibit lower *K*_d_ values toward intact cells compared to cell homogenates.

Considering the aforementioned exposure of the radioligands to intracellular components in cell homogenates, a degradation by intracellular proteases could lead to reduced radioligand concentrations and thus apparently higher *K*_d_ values. While most of the serine and cysteine proteases might be inhibited by the used inhibitor cocktail during homogenization (see Materials and Methods), a residual proteolytic activity cannot be excluded. Therefore, for the two radioligands, **[**^**64**^**Cu]Cu-NODAGA-NES2-TATE** and **[**^**64**^**Cu]Cu-NODAGA-NES5-TATE**, the stability toward cell homogenates and intact cells under assay conditions were exemplarily examined (Figure S3 in the Supporting Information). Both compounds remained comparably stable after 1 h of incubation to both receptor sources (≥87%) and thus, a proteolytic degradation might not contribute to the observed *K*_d_ shift.

### Agonists versus Antagonists

The parent SST_2_ ligand TATE is similar to octreotide or [Tyr^3^]-octreotide, an agonist of the SST_2_ receptor as previously characterized in functional assays assessing intracellular Ca^2+^-release and uptake in intact cells for different TATE derivatives.^19−21^ In line with these reports, the studied TATE derivatives herein showed a distinct uptake upon binding to SST_2_ on MPC cells revealing uptake fractions between 48 and 68% after 1 h (Table 2). In order to investigate whether the observed difference in binding affinity is agonist-specific, we functionalized the known SST_2_ antagonist **JR11**(22,23) with NODAGA and characterized its saturation binding and internalization behavior after ^64^Cu-labeling using MPC cell homogenates and intact cells (Table 2). As expected for an antagonist, the uptake fraction was significantly lower (28%) compared to the TATE derivatives. Furthermore, the *K*_d_ value of **[**^**64**^**Cu]Cu-NODAGA-JR11** was slightly lower in the cell homogenate assay compared to the intact cell assay (5.35 vs 8.17 nM), suggesting that the observed trend in the *K*_d_ values between the two sources of SST_2_ might be specific for agonists.

### Cellular Uptake and Sensitivity to Inorganic Ions

When using intact cells for radioligand binding, several aspects might complicate and affect the actual radioligand-receptor binding event.^24^ These include the following: (1) receptor-mediated cellular uptake, (2) sensitivity to inorganic ions, and (3) activity status of the receptor.

Regarding cellular uptake, we already mentioned that this process occurs in MPC cells during the time span of the saturation binding experiments (Table 2). Potentially, receptor-mediated uptake lowers the apparent dissociation constant as the number of receptor-radioligand complexes at the cell surface are lowered causing a shift in the binding equilibrium at radioligand concentrations below receptor saturation to the side of the receptor–radioligand complex. Since uptake is considerably lower for antagonists, the binding affinity should be less affected by this process. In line with this consideration, the *K*_d_ value of the antagonist **[**^**64**^**Cu]Cu-NODAGA-JR11** was similar in intact cells and cell homogenates.

Regarding the sensitivity to inorganic ions, Ribet and colleagues noted a pronounced Ca^2+^ sensitivity for the binding of ^125^I-[Tyr^11^]somatostatin to intact cells^25^ and membranes^26^ from guinea pig pancreatic acini. At low concentrations of Ca^2+^ (<100 nM), both *K*_d_ and *B*_max_ values increased 2–3-fold compared to those at optimal Ca^2+^ concentrations (>0.1 mM). In the present case, while the RPMI-1640 medium used for intact cells contains Ca^2+^ in a concentration of at least 0.4 mM,^27^ the assay buffer used for the cell homogenates contains no added Ca^2+^. Consequently, the different Ca^2+^ levels in binding assays with cell homogenates and intact cells could contribute to the observed trend in *K*_d_ values. Sodium ions are also known to regulate agonist binding to SST_2_^28^ with higher concentrations leading to a reduced binding affinity. However, the level of sodium ions in RPMI-1640 medium used for intact cells was much higher compared to the assay buffer used for cell homogenates, rendering the contribution of sodium ions to the *K*_d_ shift rather unlikely.

### Activity Status of GPCRs

As for other proteins, GPCRs pass through a dynamic equilibrium of different conformational states, including their putatively active conformation (G-protein bound state), which is affected by various factors.^29^ In terms of ligand binding, GPCRs attached to G-proteins generally exhibit a better affinity for agonists than the inactive state. A striking example for this can be found in a study of Florio and Sternweis, who observed a dramatic increase in binding potency of the agonist oxotremorine to muscarinic receptors in the presence of the G-protein *G*_0_.^30^ For adenosine A_2A_ and β_1_-adrenoreceptors, it has been shown that the preference for the G-protein coupled receptor originates on a molecular basis from a narrower binding pocket and tighter contacts to the ligand upon G-protein binding.^31,32^ For SST_2_, recent structures obtained by cryo-electron microscopy for the inactive (apo-) receptor and the G-protein/octreotide-bound receptor suggest a similar phenomenon.^33,34^ In line with this, the addition of GTP or GppNHp or pertussis toxin to membrane preparations of SST_2_-synthesizing cells led to a marked reduction in the binding affinity of different radiolabeled agonists.^35−37^ Consequently, as the GTP/GDP level might be lower in cell homogenates, the G-protein bound receptor state and thus agonist binding should actually be favored. In accordance with this consideration, Koenig et al.^38^ characterized Neuro2A neuroblastoma cells regarding the binding of the selective SST_2_ agonist [^125^I]-BIM-23027 (c[N-Me-Ala-Tyr-D-Trp-Lys-Abu-Phe]) but were not able to determine both *K*_d_ and *B*_max_ values when using intact cells, which was related by the authors to high nonspecific binding but also the presence of a high GTP concentration in the cells. Moreover, Gerwins et al.^39^ demonstrated in competition experiments that adenosine agonists bind to the adenosine A_1_ receptor in membrane preparations according to a two-site model (low and high affinity site), while only a low affinity site could be detected upon binding to intact cells. The addition of GTP (100 μM) to the membrane preparations shifted the curve pattern to a one-site model and the obtained *K*_d_ values were in agreement with the *K*_d_ values obtained with intact cells. However, the results herein are in contrast to the aforementioned studies as the binding of the radioligands was even more favorable toward intact cells compared to cell homogenates. This could point to the fact that the SST_2_ receptor in the MPC cells is in a large excess over the respective G-protein(s), rendering the fraction of G-protein-bound receptor less significant according to theoretical considerations.^40,41^

When discussing differences in ligand binding between different receptor sources, one should also consider the significance of the determined dissociation constants. There is the general view that measuring the binding affinity of GPCR agonists is always affected by the efficacy of the agonists,^40,42^ which includes amongst others receptor-mediated uptake and G-protein binding. This brings us to the point that the applied assay conditions for neither the cell homogenates nor the intact cells might provide experimentally determined dissociation constants (also named *K*_obs_) according to the classic definition of the dissociation constants (*K*_d_) since G-protein binding among others was not prevented from the outset. However, considering that, e.g., receptor-mediated uptake of radiolabeled SST_2_ agonists also occurs in vivo,^43,44^ intact cells might represent, from our perspective, a better physiological model for the evaluation of the radioligand performance. We would also like to point out in this context, that after ^64^Cu-labeling of the TATE derivatives, separation of the non-labeled ligand was not performed. Consequently, the data shown in Table 2 are obtained for a mixture of ^64^Cu-labeled and non-labeled ligands with apparent molar activity^45^ values in the range of 20–50 GBq/μmol. We are aware that this certainly led to an altered binding affinity compared to the pure metal complexes as previously shown by Reubi et al.^46^ However, the separation of radioligands is typically not required for preclinical and clinical applications (provided the molar activity is sufficiently high). Therefore, we have omitted radioligand separation for the in vitro binding experiments.

## Conclusions

Overall, the use of intact MPC cells instead of cell homogenates provides an appropriate assay for assessing the binding properties of newly developed ligands to SST_2_. The tendency in binding affinities appears conserved between cell homogenates and intact cell, but the nonspecific binding to intact cells is markedly reduced compared to the nonspecific binding to cell homogenates. The general trend to lower binding affinities toward intact cells cannot be fully rationalized but appears to be agonist-specific and might arise from several aspects, with the concurrent cellular uptake and the potential Ca^2+^ sensitivity of SST_2_ being just two potential explanations. The pharmacological effects of agonist binding toward SST_2_ on MPC cells will be further explored complemented by comparisons to human cells with a high abundance of SST_2_.

## Materials and Methods

### Radiolabeling of DOTA/NODAGA-Bearing Peptides^16,17^

[^64^Cu]CuCl_2_ was produced at the Helmholtz-Zentrum Dresden-Rossendorf on the 30 MeV TR-Flex-Cylotron (Advanced Cyclotron Systems Inc., ACSI, Canada) by a ^64^Ni(p,n)^64^Cu nuclear reaction as reported previously.^47,48^

For a typical radiolabeling procedure, 550 MBq of [^64^Cu]CuCl_2_ (60 μL in H_2_O) was mixed with 0.01 M HCl (230 μL) and ammonium acetate buffer (2 M, pH 8, 30 μL) to obtain a solution with a pH value around 5.5. An aliquot of this mixture (105 μL) was then added to the peptide stock solution (2.5 μL of 2 mM in 10% DMSO/PBS, pH 7.4), and the mixture was incubated for 20 min at 60–80 °C. Quality control of the radiolabeled peptide conjugates was performed by radio-HPLC analysis.^16,17^ Labeling yields were usually ≥97%. The radiolabeled peptides were used without purification. Molar activities of up to 50 GBq/μmol were achieved and were calculated based on the applied peptide amount. For further binding experiments, the reaction mixture was diluted with cell culture medium or phosphate buffered saline (PBS, pH 7.4).

### In Vitro SST_2_ Binding Affinity Using MPC Cell Homogenates^16^

MPC cells (passages 35–40) were routinely cultured in collagen-coated flasks as described elsewhere^49^ and harvested at 70–80% confluency in Dulbecco’s phosphate-buffered saline containing 2.0 mM ethylenediaminetetraacetic acid (EDTA) at 4 °C for 30 min. Cells were resuspended and frozen in fetal bovine serum containing 10% (v/v) DMSO and stored at −70 °C. After thawing, cells were washed and resuspended in ice-cold saturation assays buffer, pH 7.4, containing 50 mM Tris-HCl, 1 mM EDTA, 0.5 mM *o*-phenanthroline, and 0.1% (w/v) bovine serum albumin. Cells were homogenized in ice-cold saturation assay buffer supplemented with complete EDTA-free proteinase inhibitor (Roche, Basel Switzerland) using a Dounce homogenizer. Protein content of cell homogenates was measured at *A*_280nm_ (setting 1 Abs = 1 mg/mL) using a nanodrop spectrophotometer (Thermo-Fisher Scientific).

For the measurement of total binding, 0.155 mL of cell homogenates were incubated with radioligands (molar activity (*A*_m_) = 25 MBq/nmol) at increasing final concentrations between 0.625 and 40 nM (final sample volume 0.2 mL) in Polystyrene tubes (5 mL, round bottom, clear, Greiner bio-one, Item no. 115101). For the measurement of nonspecific binding, specific binding sites were saturated with non-labeled DOTA-TATE at a final concentration of 1 μM. Samples were incubated for 60 min at 37 °C. Incubation was stopped by soaking cell homogenates into Whatman GF/C collection filters (GE Healthcare, Chicago, IL, USA; presoaked in 0.3% (v/v) polyethyleneimine for 90 min) and washing with ice-cold Dulbecco’s phosphate-buffered saline using a cell harvester (Brandel, Gaithersburg, MD, USA). Nonspecific binding of the radioligands (at 0.312, 1.25, 5, and 20 nM, with and without 1 μM of non-labeled DOTA-TATE; the values at the other radioligand concentrations were derived by linear regression) to the filters was assessed in the absence of cell homogenates. Activity bound to filters was measured using the gamma counter Wizard (PerkinElmer). Activity in a series of radioligand standards was measured at increasing molar amounts between 0.625 and 40 nM. Measurements for total binding were performed in duplicate, while single measurements were performed for nonspecific binding.

### In Vitro SST_2_ Binding Affinity Using Intact MPC Cells^17^

A number of 3 × 10^5^ cells/cm^2^ were seeded in collagen-coated 48-well microplates (CELLSTAR 48 Well Cell Culture Multiwell Plates, Polystyrene, Greiner bio-one, Item no. 677180) and grown for three days. For binding assays, cell culture medium was removed and replaced by fresh medium supplemented with the radioligand (*A*_m_ = 25 MBq/nmol) at increasing final concentrations between 0.321 and 40 nM (final sample volume 0.2 mL). Nonspecific cell binding was measured in the presence of non-labeled DOTA-TATE at a final concentration of 1 μM. Nonspecific binding to plastic surfaces was determined in cell-free wells at radioligand concentrations of 0.312, 1.25, 5, and 20 nM (with and without 1 μM non-labeled DOTA-TATE; the values at the other radioligand concentrations were derived by linear regression). Samples were incubated for 60 min at 37 °C. Incubation was stopped by washing with ice-cold Dulbecco’s PBS. Cells were lysed with 0.1 M NaOH containing 1% (w/v) SDS. Activity was measured in cell homogenates and in a series of radioligand standards containing increasing molar amounts between 0.06 and 8 pmol using the gamma counter Wizard (PerkinElmer). Protein content of cell homogenates was measured as described above. Measurements for total binding were performed in triplicate, while measurements for nonspecific binding were performed in duplicates.

### Determination of *K*_d_ and *B*_max_ Values

Plots of “total binding” = *f*(radioligand) were analyzed by nonlinear regressions using the model of “one site—total, accounting for ligand depletion” as implemented in GraphPad Prism and Plots of “nonspecific binding” = *f*(radioligand) were analyzed by linear regressions. For the ligand depletion model, the term “NS” was constrained to the respective slopes obtained by the linear regressions. The terms “SpecAct” (obtained with standard curves) and “Vol” (0.2 mL, assay volume for both SST_2_ sources) were also constrained. *K*_d_ values were derived in nM, and the *B*_max_ values (in cpm) were transformed into fmol/mg. Both data sets were corrected for nonspecific binding, i.e., binding to the Whatman GF/C collection filters (in the absence of cell homogenate) or binding to the microplate cavities (in the absence of intact cells).

### Cell Binding and Uptake

MPC cells were seeded into collagen-coated 24-well microplates and cultivated for 4 days. All washing steps were performed using PBS containing 0.9 mM CaCl_2_ and 0.5 mM MgCl_2_. Total radioligand uptake was measured after incubation with the radioligand (*A*_m_ = 30 GBq/μmol) at a final concentration of 20 nM in RPMI 1640 medium with GlutaMAX supplement (Thermo Fisher Scientific) for 1 h at 37 and 4 °C. Nonspecific binding was determined in the presence of 20 μM Acetyl-TATE. The uptake fraction was measured after acid wash of cell surface-bound radioligand with wash buffer containing 0.05 M glycine, pH 2.8, for 5 min. The activity of cell homogenates was measured using the γ counter Wizard (PerkinElmer). The protein content of cell homogenates was measured as described above.

## Supporting Information Available

Synthesis and characterization of NODAGA-JR11 and [^64^Cu]Cu-NODAGA-JR11 saturation binding curves for the ^64^Cu-labeled TATE derivatives and [^64^Cu]Cu-NODAGA-JR11; comparison of assay settings for [^64^Cu]Cu-NODAGA-NES5-TATE; stability toward cell homogenate and intact cells; and cell binding data for the ^64^Cu-labeled TATE derivatives (PDF)

## Notes

The TOC graphic was created with biorender.com.
