# Supplementary material for: Comparative Saturation Binding Analysis of 64Cu-Labeled Somatostatin Analogues Using Cell Homogenates and Intact Cells
Source: ACS Omega. 2023 Jun 22;8(26):24003–9. doi: 10.1021/acsomega.3c02755 (PMC10324063; doi:10.1021/acsomega.3c02755)
Supplement: Supplementary file 1 — ao3c02755_si_001.pdf [file ao3c02755_si_001.pdf]

Supporting Information

Comparative saturation binding analysis of ^{64}Cu-labeled somatostatin analogs using cell homogenates and intact cells

Martin Ullrich[a,*], Florian Brandt[a,b], Reik Löser[a,b], Jens Pietzsch[a,b], Robert Wodtke[a,*]

[a] Helmholtz-Zentrum Dresden-Rossendorf, Institute of Radiopharmaceutical Cancer Research, Bautzner Landstraße 400, 01328 Dresden, Germany

[b] Technische Universität Dresden, School of Science, Faculty of Chemistry and Food Chemistry, Mommsenstraße 4, 01069 Dresden, Germany

*E-mail: m.ullrich@hzdr.de; r.wodtke@hzdr.de

Table of Contents

Synthesis and characterization of NODAGA-JR11 3

Characterization of [^{64}Cu]Cu-NODAGA-JR11 4

Figure S1: **Saturation binding curves for the ^{64}Cu-labeled TATE derivatives and [^{64}Cu]Cu-NODAGA-JR11 .... 5**

Figure S2: **Comparison of assay settings for [^{64}Cu]Cu-NODAGA-NES5-TATE 8**

Figure S3: **Stability toward cell homogenate and intact cells 9**

[^{64}Cu]Cu-NODAGA-NES2-TATE 9

[^{64}Cu]Cu-NODAGA-NES5-TATE 10

Figure S4: **Cell binding data for the ^{64}Cu-labeled TATE derivatives.......... 11**

References 12

Synthesis and characterization of NODAGA-JR11

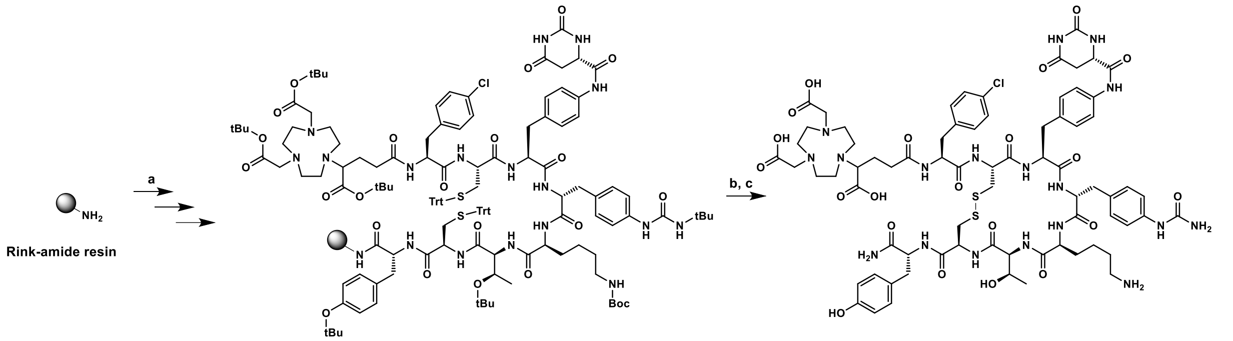

NODAGA-Cpa-c(D-Cys-Aph(Hor)-D-Aph(Cbm)-Lys-Thr-Cys]-D-Tyr-NH_2

NODAGA-JR11 was synthesized according to previously published procedures.[1, 2] In brief (Scheme above): **a**) Fmoc/*t*Bu-based solid-phase peptide synthesis using an automated microwave peptide synthesizer (Biotage Initiator+ Alstra) with standard protocols for coupling (HATU/DIPEA) and Fmoc removal (20% piperidine/DMF) **b**) TFA/TIPS/H_2O (95:2.5:2.5, v/v/v); 40°C, 4 h; **c**) 10% DMSO in CH_3CN/H_2O (25:75, v/v), pH 8.0, 48-72 h.

After purification by RP-HPLC, NODAGA-JR11 (8.6 mg, 9%) was obtained as a white fluffy solid. HRMS (ESI+) 830.3097 calculated for $[M+2H]^{2+}$, found 830.3107. Purity (≥95) was determined by analytical RP-HPLC at 230 nm.

HRMS spectrum

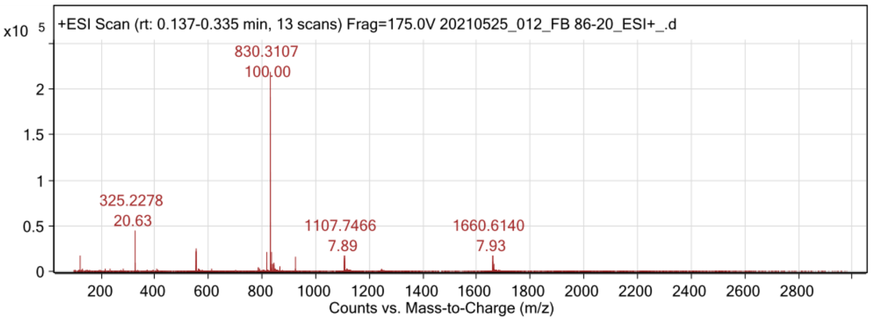

Peak List

m/z	z	Abund
325.2278	1	45167.67
553.8765	1	24945.78
554.2109	1	22636.57
816.4073	1	20649.35
830.3107	2	218905
830.8121	2	190693.77
831.3114	2	176811.48
831.8117	2	108830.39
832.312	2	53193.43
832.8122	2	21457.39

Characterization of [^{64}Cu]Cu-NODAGA-JR11

An exemplary radio-HPLC chromatogram for **[^{64}Cu]Cu-NODAGA-JR11** is shown below after labeling with copper-64.

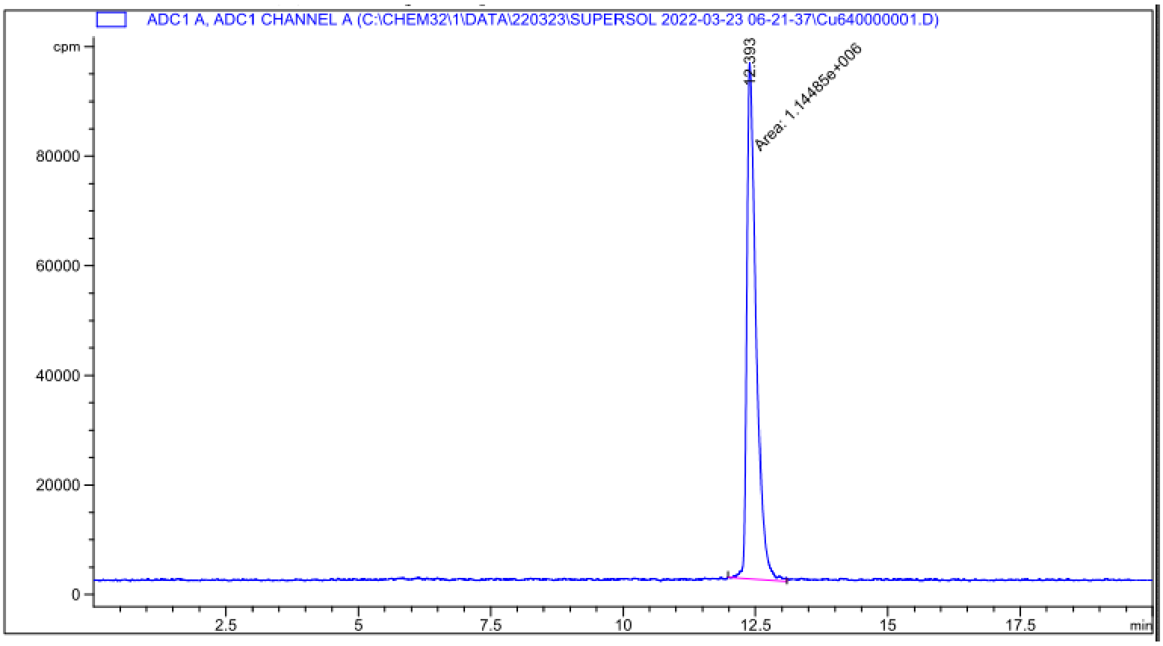

Peak #	RetTime [min]	Type	Width [min]	Area [cpm*s]	Height [cpm]	Area %
1	12.393	MM	0.2026	1.14485e6	9.41579e4	100.0000
Totals :				1.14485e6	9.41579e4	

Figure S1: Saturation binding curves for the ^{64}Cu-labeled TATE derivatives and [^{64}Cu]Cu-NODAGA-JR11

Saturation binding toward MPC cell homogenates (left column) and intact cells (right column) with data for total and nonspecific binding (in the presence of 1 µM DOTA-TATE) shown as filled and open circles, respectively. Regression analysis was performed by using the model of “One site – total and nonspecific binding” as implemented in GraphPad Prism (Version 9.4.1).

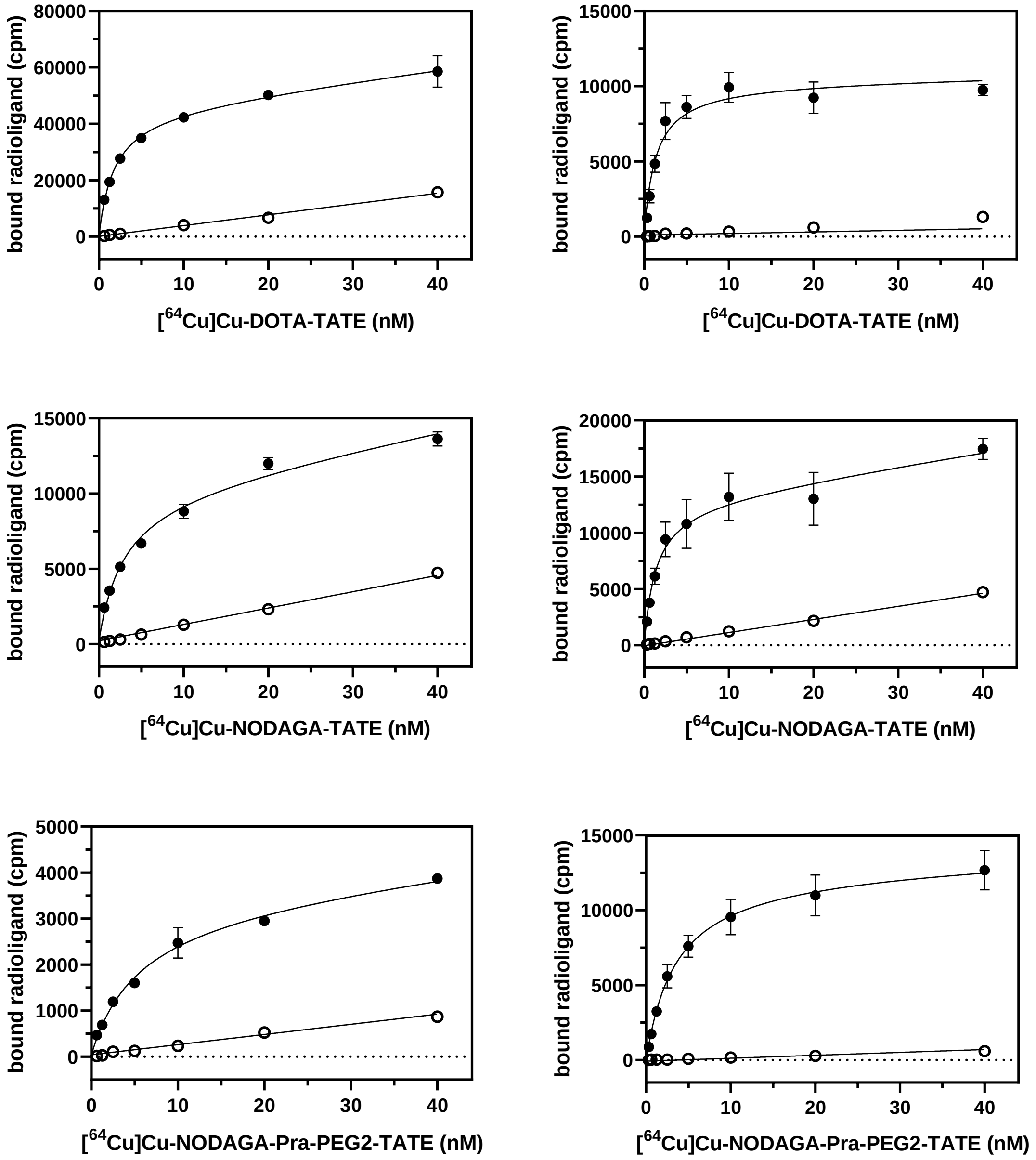

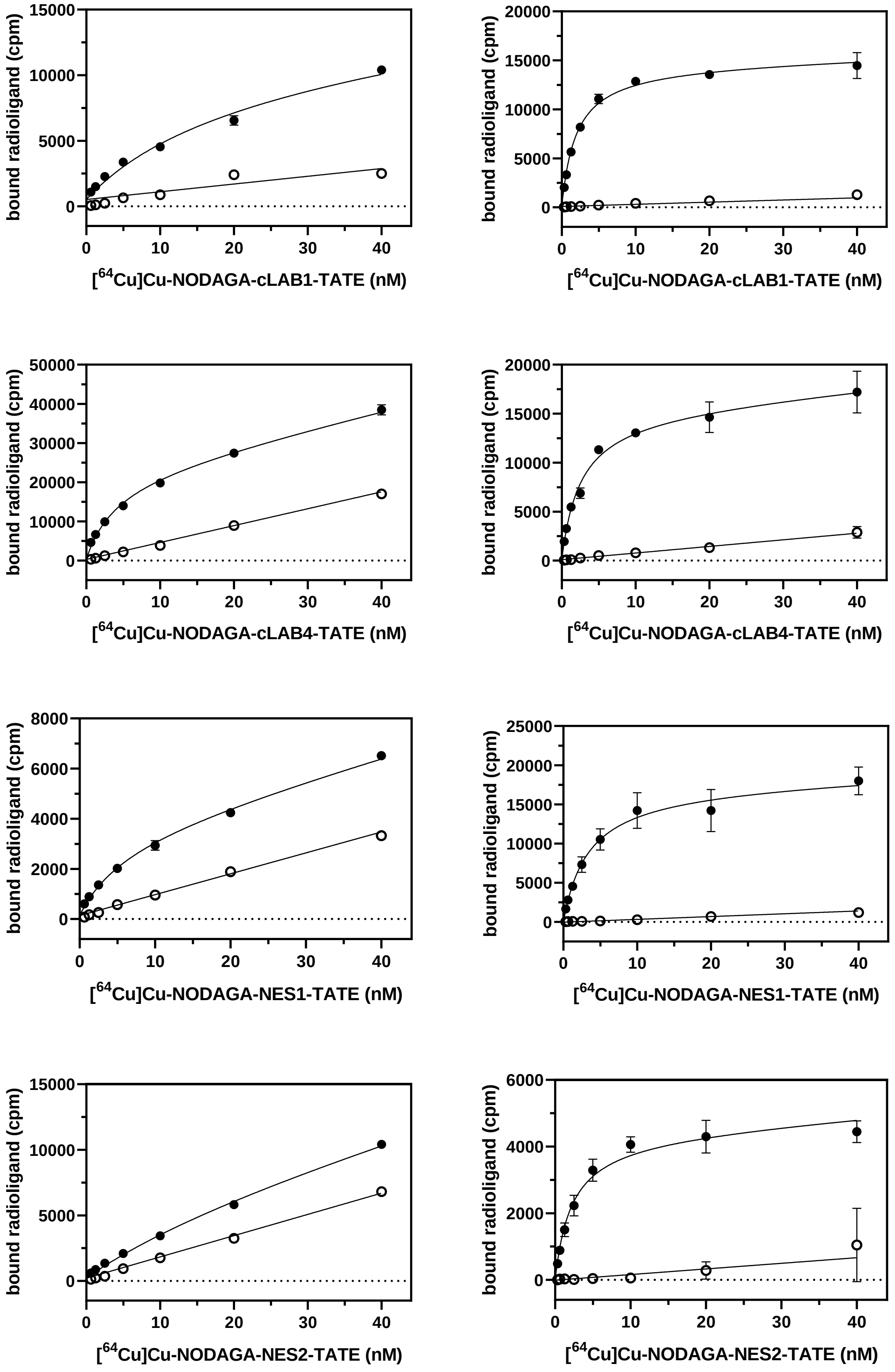
bound radioligand (cpm)
15000
10000
5000
0
0
10
20
30
40
[^{64}Cu]Cu-NODAGA-cLAB1-TATE (nM)
bound radioligand (cpm)
20000
15000
10000
5000
0
0
10
20
30
40
[^{64}Cu]Cu-NODAGA-cLAB1-TATE (nM)
bound radioligand (cpm)
50000
40000
30000
20000
10000
0
0
10
20
30
40
[^{64}Cu]Cu-NODAGA-cLAB4-TATE (nM)
bound radioligand (cpm)
20000
15000
10000
5000
0
0
10
20
30
40
[^{64}Cu]Cu-NODAGA-cLAB4-TATE (nM)
bound radioligand (cpm)
8000
6000
4000
2000
0
0
10
20
30
40
[^{64}Cu]Cu-NODAGA-NES1-TATE (nM)
bound radioligand (cpm)
25000
20000
15000
10000
5000
0
0
10
20
30
40
[^{64}Cu]Cu-NODAGA-NES1-TATE (nM)
bound radioligand (cpm)
15000
10000
5000
0
0
10
20
30
40
[^{64}Cu]Cu-NODAGA-NES2-TATE (nM)
bound radioligand (cpm)
6000
4000
2000
0
0
10
20
30
40
[^{64}Cu]Cu-NODAGA-NES2-TATE (nM)

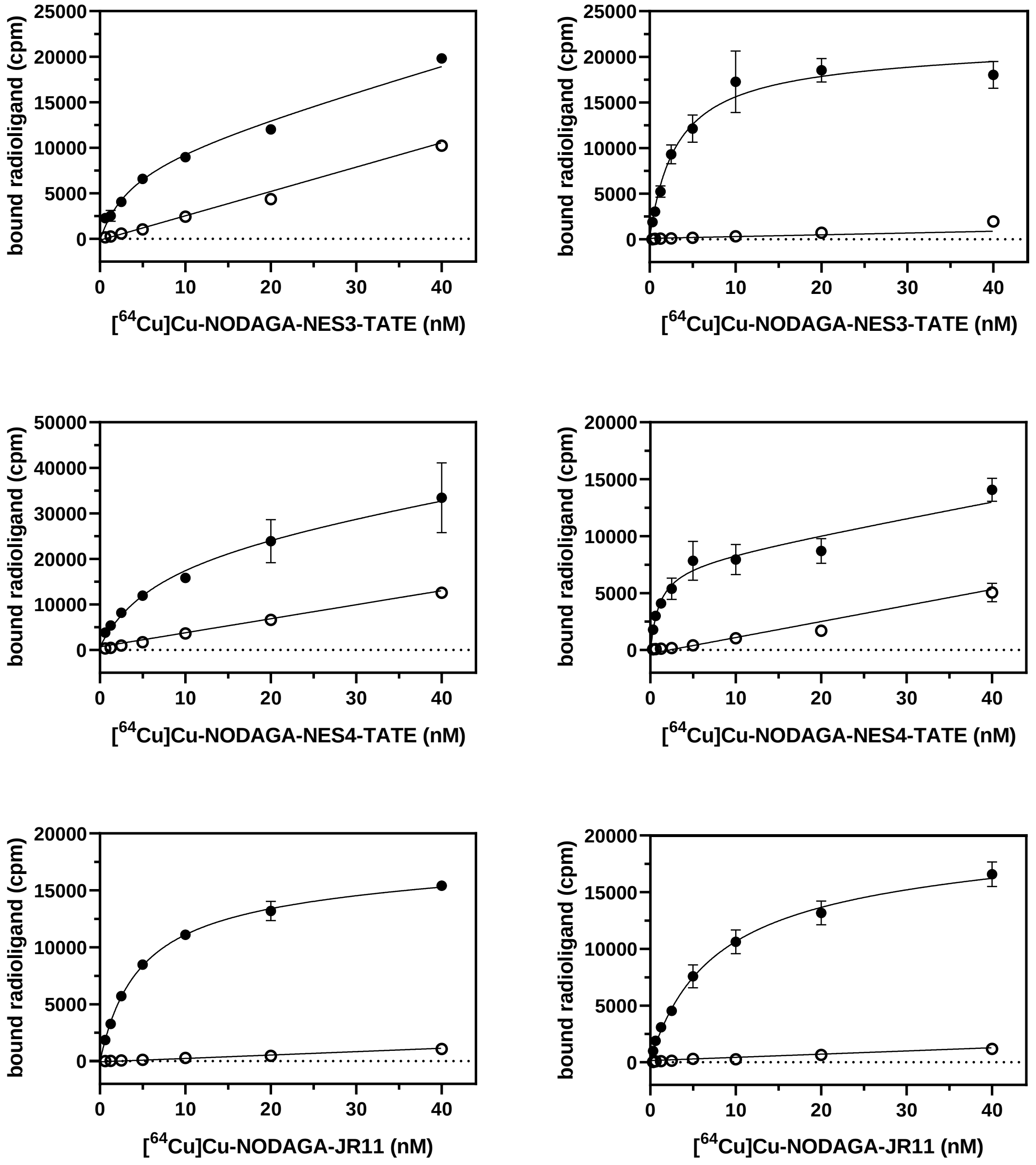
bound radioligand (cpm)
[64Cu]Cu-NODAGA-NES3-TATE (nM)
[64Cu]Cu-NODAGA-NES4-TATE (nM)
[64Cu]Cu-NODAGA-JR11 (nM)

Figure S2: Comparison of assay settings for [^{64}Cu]Cu-NODAGA-NES5-TATE

Saturation binding of **[^{64}Cu]Cu-NODAGA-NES5-TATE** toward intact cells (adherent in 48-well plates), MPC cell homogenates and intact cells in suspension with data for total and nonspecific binding (in the presence of 1 µM Acetyl-TATE) shown as filled and open circles, respectively. All data were obtained at the same day with the same preparation of the radioligand and were corrected for binding to the wells or filters. Moreover, the protein amount in each setting was comparable (0.15-0.2 mg per incubation). Regression analysis was performed by using the model of "One site – total and nonspecific binding" as implemented in GraphPad Prism (Version 9.4.1). The following K_d and B_{max} values (68% CI) were derived: 1.06 nM (0.93-1.20) and 203 fmol/mg (198-209) for adherent intact cells, 13.5 nM (9.92-18.8) and 919 fmol/mg (811-1062) for cell homogenates, 1.90 nM (1.43-2.49) and 733 (684-784) fmol/mg for intact cells in suspension. The data show that the nonspecific binding of the radioligand is significantly higher in case of cell homogenates in comparison to intact cells using the same assay setting (cell harvester). Therefore, the radioligand binds most likely to cell components in the cell homogenates that are not accessible in intact cells highlighting the advantage of using intact cells for saturation binding from a methodological perspective as also discussed in the main article.

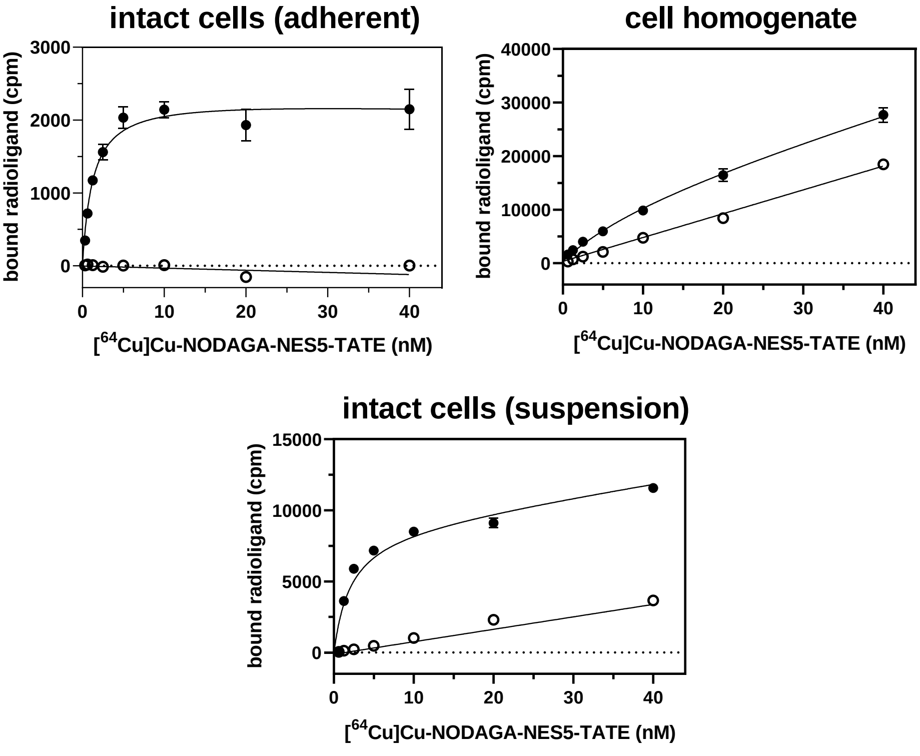

Figure S3: Stability toward cell homogenate and intact cells

[^{64}Cu]Cu-NODAGA-NES2-TATE and **[^{64}Cu]Cu-NODAGA-NES5-TATE** (40 nM) were incubated at 37°C under assay conditions in the presence of MPC cell homogenate or MPC intact cells. After 1 h, an aliquot (160 µL) was withdrawn, mixed with “Supersol” (320 µL), which consists of 20% ethanol, 0.5% Triton X-100, 5 mM EDTA, 0.5 mM o-phenanthroline, and 0.1% saponin. This was followed by centrifugation at 13,500 rpm for 3 min (Thermo Scientific Heraeus Fresco 21). The supernatant was analyzed by radio-HPLC.[1, 2]

[^{64}Cu]Cu-NODAGA-NES2-TATE

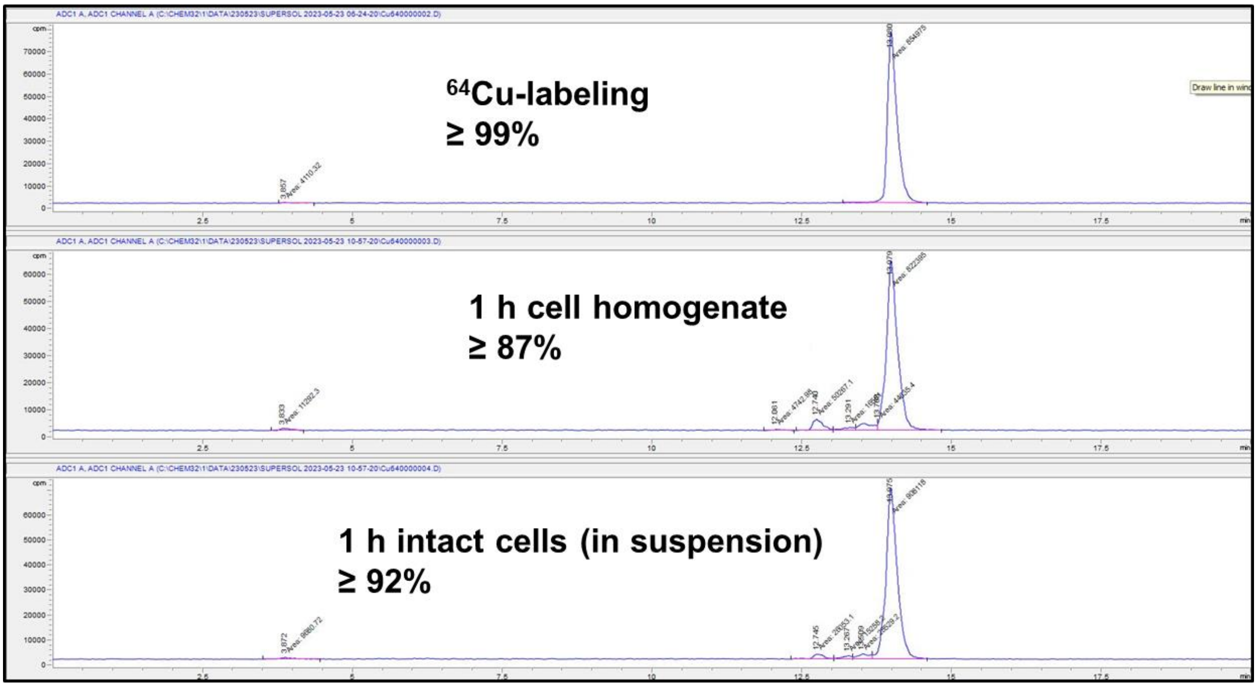

[^{64}Cu]Cu-NODAGA-NES5-TATE

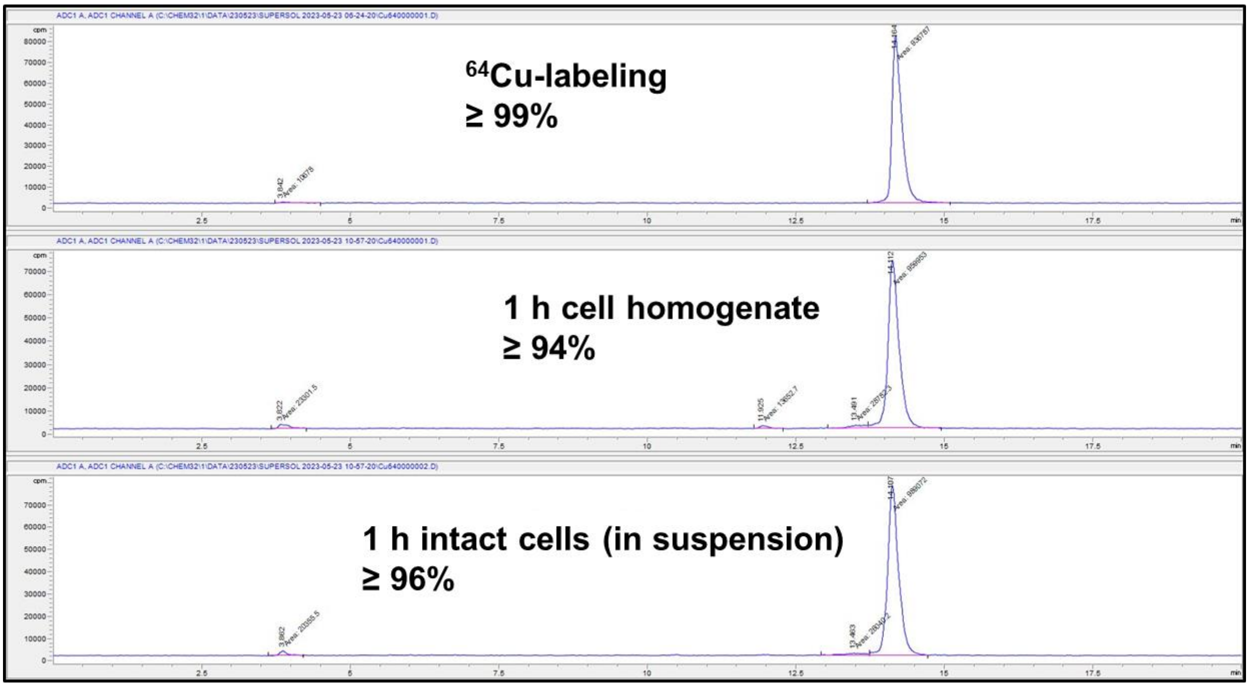

Figure S4: Cell binding data for the ^{64}Cu-labeled TATE derivatives[1]

Nonspecific binding was determined in the presence of 20 µM Acetyl-TATE (radioligand concentration of 20 nM). Data shown in **A** and **B** are mean values (±SD) of one experiment, which was performed in sextuplicate. In **C**, the uptake fraction ("internalized" or not acid releasable radioligand, red bars in **A**, mean values) is expressed as percentage of specific total-bound radioligand (blue bars in **A**, mean values). The difference to 100% was assigned to "surface-bound" radioligand. For a better overview, the compound names were abbreviated.

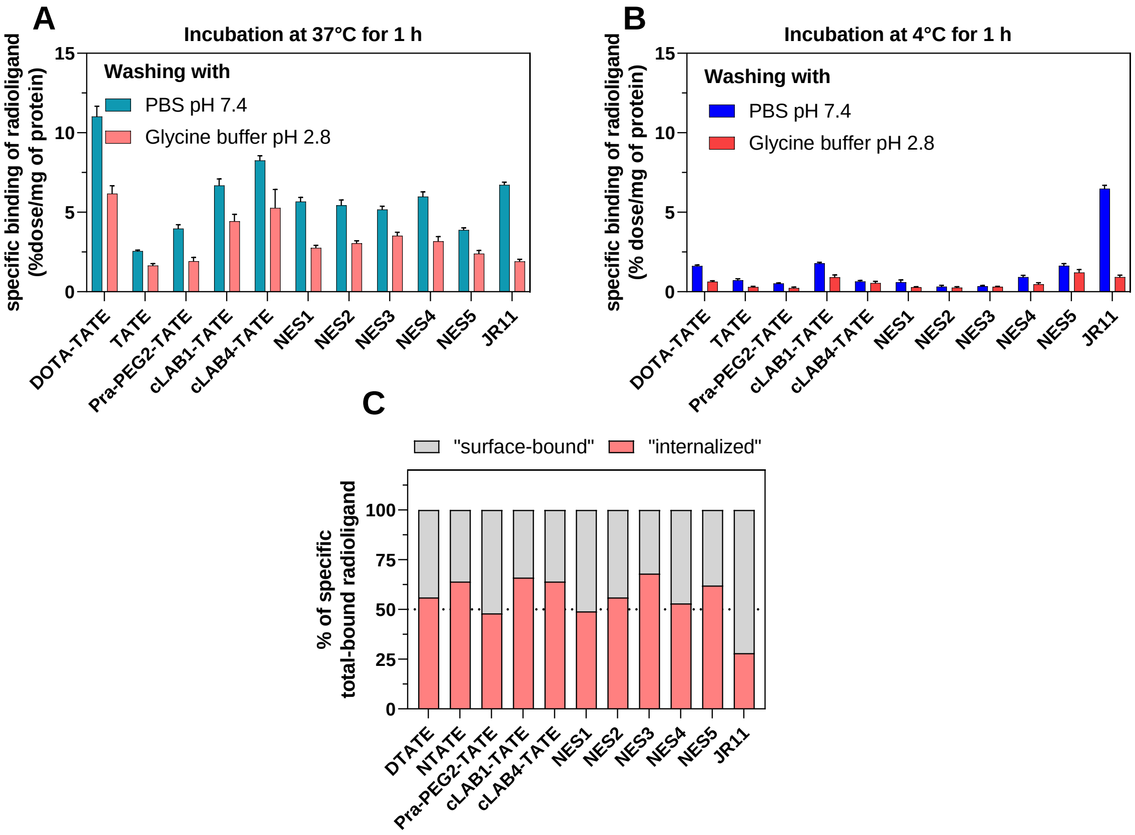

References

1. Brandt, F.; Ullrich, M.; Wodtke, J.; Kopka, K.; Bachmann, M.; Löser, R.; Pietzsch, J.; Pietzsch, H. J. and Wodtke, R. Enzymological Characterization of ^{64}Cu-Labeled Neprilysin Substrates and Their Application for Modulating the Renal Clearance of Targeted Radiopharmaceuticals. *J. Med. Chem.*, **2023**, *66*, 516-537.
2. Brandt, F.; Ullrich, M.; Laube, M.; Kopka, K.; Bachmann, M.; Löser, R.; Pietzsch, J.; Pietzsch, H. J.; van den Hoff, J. and Wodtke, R. "Clickable" albumin binders for modulating the tumor uptake of targeted radiopharmaceuticals. *J. Med. Chem.*, **2022**, *65*, 710-733.
